# Association between social determinants of health and direct economic burden on middle-aged and elderly individuals living with diabetes in China

**DOI:** 10.1371/journal.pone.0250200

**Published:** 2021-04-15

**Authors:** Jianwei Guo, Yun Wu, Xinlei Deng, Zifeng Liu, Lijin Chen, Yixiang Huang

**Affiliations:** 1 Department of Health Policy and Management, School of Public Health, Sun Yat-sen University, Guangzhou, Guangdong, P. R. China; 2 Department of Environmental Health Sciences, University at Albany, the State University of New York, Rensselaer, New York, United States of America; 3 Department of Clinical Data Center, the 3rd Affiliated Hospital, Sun Yat-sen University, Guangzhou, China; Jatiya Kabi Kazi Nazrul Islam University, BANGLADESH

## Abstract

**Aims:**

The aim of this study was to determine the association between social determinants of health and direct economic burden on Chinese middle-aged and elderly individuals living with diabetes in China.

**Methods:**

This study used data from the baseline wave of The China Health and Retirement Longitudinal Study (CHARLS) database, covering 17,708 middle-aged and elderly residents in China. The population with diabetes was grouped into those diagnosed with diabetes mellitus (DDM) and those undiagnosed with diabetes mellitus (UDM). Direct economic cost data, including total direct medical costs (TC) and out-of-pocket (OOP) payments, were extracted as outcome variables. A two-part model was applied to analyze the association between social determinants of health and direct economic burden.

**Results:**

In our analysis, we included 958 patients with DDM and 1,285 patients with UDM. The mean TC and OOP payments were 11,193 CNY (US $1,733; 6.46 CNY = 1 USD) and 7,266 CNY (US $1,125) in DDM patients, and 3,700 CNY (US $573) and 3,060 CNY (US $474) in UDM patients. Rural-urban status (p<0.05), regional status (p<0.05), household personal consumption expenditures (p<0.05), and comorbidities(p<0.05) were crucial factors associated with medical costs in people with diabetes.

**Conclusion:**

Although progress has been made in the development of current health policies intended to contain the direct economic burden of diabetes, the gaps in that burden in populations with different social characteristics remains a burning issue. More policy breakthroughs are needed to achieve health equity.

## Background

Diabetes mellitus, with a prevalence of 8.5% in the world population, is the eighth leading cause of deaths in the world [1]. China ranks second world-wide in diabetes prevalence, with one quarter of the world’s more than one hundred million patients [2, 3]. The occurrence of diabetes in China is positively related to aging, with a prevalence lower than 3.0% in people younger than 45 years, 11.5% in the middle-age group (ages 45–59 years), and more than 20% in the elderly (ages ≥60 years) [4, 5]. Since diabetes is a life-long chronic disease, affected individuals suffer greater risk of developing comorbidities and complications, such as cardiovascular diseases, physical dysfunction, mental disorders, etc. [6–8]. Half of the individuals with diabetes have at least one comorbid condition, while the situation is more severe in middle-aged and elderly patients [7]. The pathophysiological nature of diabetes leads to a vastly increased need and demand for medical services, thereby causing high diabetes-related healthcare expenditures [8–12]. Since China had over 280 million people aged ≥ 60 years in 2020, research on factors influencing diabetes-related costs is crucial for the development of mechanisms to help the country cope with the booming economic burden faced by individuals with diabetes.

Social determinants of health, defined as the conditions in which people are born, live, work, and age that affect health outcomes, have been found to play considerable roles in affecting the economic burden of diabetes [13–15]. Regional residential-related differences, including different political situations and healthcare systems and many other societal components, are crucial factors determining the prevalence and healthcare costs of diabetes [16]. Among these factors, poverty and income level have been recognized as the key determinants affecting the economic burden of diabetes. On the one hand, poverty might be one of the sources of poor prevention of diabetes, leading diabetes-related costs to rise as diabetes-related comorbidities and complications progress [16, 17]. Conversely, patients with higher income levels tend to be able to afford more expensive and longer-term therapeutic strategies, resulting in higher explicit costs [18]. Similar to those living in poverty, people with lower education levels usually have more frequent complications of diabetes due to negligence in disease prevention [19]. In China, the association between social determinants of health and economic burden of diabetes appears to be more profound and complicated. Residential segregation in particular, including rural-urban and internal regional has been proposed as a crucial social determinant given the appreciable socio-economic differences among regions. However, there is no current evidence to reveal the association between regional residential status and economic burden in patients with diabetes. Additionally, the types of medical insurance, occupations, and work status, and marital status have also been associated with the economic burden of diabetes, but their mechanisms have not yet been articulated [20–22].

In pursuit of the goal of Universal Health Coverage put forward by the World Health Organization and Sustainable Development Goals put forward by the United Nations, studies are needed to guide health actions to contain the growth of the economic burden of diabetes and prevent the occurrence of catastrophic healthcare expenditures [1, 23, 24]. However, current studies either focused only on developed countries [8, 9, 11, 12, 19] or drew conclusions based on specific domestic regions (e.g. a province or a city) or populations (e.g., a hospital-based population or an insured population) in China [21, 22], which make them less convincing for national policy-making. In addition, social determinants were mostly studied as factors subordinate to clinical factors in the formation of diabetes-related costs so that their immediate association with the economic burden of diabetes is often vague [8, 10–12, 18]. Therefore, the aims of this study were (1) to reveal the direct economic burden on middle-aged and elderly individuals with diabetes in China and (2) to study how social determinants of health are associated with direct economic costs in these individuals.

## Methods

### Data sources

This study used data from The China Health and Retirement Longitudinal Study (CHARLS) database. CHARLS is a nationally representative population-based survey designed to study social, health, and economic issues of residents aged ≥ 45 years in light of the burgeoning aging population in China [25]. Using the Probabilities Proportional to Size (PPS) sampling method, by adopting regional status, urban-rurality, and gross domestic product data in 2009 as stratified indicators, CHARLS followed the top-down county-neighborhood-household-individual order procedure in sampling. For sample weighting, CHARLS constructed sample weights for households, individuals, and biomarker data directly from the sampling probabilities [25]. Face-to-face computer-assisted personal interviews (CAPI), physical measurements, and blood tests were conducted every 2 years to collect data. This study used data from the baseline 2011 wave conducted between June 2011 and March 2012, covering a total of 17,708 residents living in 10,257 households across 28 provinces in China. The study excluded 396 residents who refused to be interviewed or did not finish the survey, 360 respondents under 45 years of age, and 168 respondents who did not take the blood test. Ultimately, 16,784 respondents were included in our study.

### Definitions

The diagnostic criteria for diabetes mellitus recommended by the American Diabetes Association in 2011 were adopted to define the population with diabetes [26]. During the CAPI personal interviews, respondents were asked the question, “Have you been diagnosed with diabetes?”, and evidence for diagnosis such as blood test reports (which reported a fasting glucose ≥ 126 mg/dl, or HbA1c ≥ 6.5%, or HbA1c ≥ 48 mmol/mol) previous to the CHARLS survey was requested as well. People who affirmed that they had been diagnosed with diabetes were defined as ‘diagnosed diabetes mellitus’ (DDM). Those who denied a history of diagnosed diabetes but reported a fasting glucose ≥ 126 mg/dl or HbA1c ≥ 6.5% (HbA1c ≥ 48 mmol/mol) in their blood test were defined as ‘undiagnosed diabetes mellitus’ (UDM).

### Costs measurement

In this study, the direct economic burden consisted of direct medical costs and direct nonmedical costs. Direct medical costs included costs such as medications and tests generated by outpatient visits, inpatient visits, and self-health care. Direct nonmedical costs included the costs of transportation to healthcare providers, visits by relatives and/or friends during inpatient and outpatient periods, accommodations, and food. Monthly or annual costs were requested during the interview, and we calculated the annualized total direct cost (TC) by multiplying the monthly costs by 12, or equal to the annual costs recalled. The out-of-pocket (OOP) payments were also requested and recorded. We did not include indirect costs for analysis because our study population consisted of middle-aged and elderly individuals, some of whom were retired or receded and were unable to provide accurate recall, so that indirect costs were hard to measure [27]. Costs were presented in Chinese Yuan (CNY, 6.46 CNY = 1 USD in 2011).

### Study variables

In our study, we focused on medical costs as the outcome variable under the morbid conditions of diabetes. Variables of social determinants included gender, age (categorized into age groups of 45–54, 55–64, 65–74, and ≥ 75 years), rural-urban status, regional location (categorized into east, middle, and west China), marital status, highest completed education level, occupations (categorized into unemployed/unpaid, agricultural work, employed, self-employed, retired/receded), household personal consumption expenditures (averaged into 5 levels), medical insurance, and comorbidities. In this study, we used household personal consumption expenditures instead of household income or personal income because a previous study showed that the former variable was more responsive for distinguishing the catastrophic effect of healthcare costs [28].

### Statistical analysis

We used multiple imputations to cope with missing data. Variables which contained missing data included missing days of work (23/16,784; with a missing rate of 0.1%) and whether they had bought additional insurance (40/16,784; with a missing rate of 0.2%), which were not included in our study variables. We imputed these variables for further logic inspection of the value of the study variables (including occupation and medical insurance) to confirm its accuracy. Characteristics of patients with DDM and UDM were reported by subgroups of demographics. The statistically significant differences by groups were examined with a chi-square test. Medical costs, including TC and OOP payments, in the population with DDM and UDM are presented as median and inter-quartile range as well as mean and standardized error by subgroups of demographics and social determinants. Univariate non-parametric tests were conducted to examine statistical significance among groups. For multivariate analyses, a mixed-effect two-part model was applied: In the first part, logistic regressions were used to analyze the associations between social determinants and the incurrence of medical costs; in the second part, generalized linear mixed models were used to analyze the associations between social determinants and the quantitative level of costs. To satisfy the requirement of generalized linear models, data of samples with non-zero costs were logarithmically transformed to be normally distributed. Compared with a medical-record-based design, a population-based design study usually includes patients who do not spend directly for their health even when they clearly know that they are suffering from diseases. By using the two-part model, we could study not only the association between healthcare cost levels and social determinants of health but also the association between the healthcare costs incurred and our study variables. The level of statistical significance was set as p value = 0.05. Data cleaning and statistical analyses were conducted with Stata V.15.0 (StataCorp, College Station, Texas, USA).

## Results

### Prevalence of diabetes and characteristics of the study population

Among the 16,784 respondents included in our study, there were 958 patients with DDM and 1,285 patients with UDM, at a prevalence of 5.71% and 7.66%, respectively. Characteristics of the study population are summarized in Table 1. Among the population with diabetes, the majority with DDM were female (p = 0.004), aged 55–74 (p = 0.045), live in urban areas (p<0.001), live in the east (p<0.001), have completed higher education levels (p<0.001), have been retired/receded (p<0.001), have higher levels of household consumption expenditures (p<0.001), and have more kinds of comorbidities, but to show no differences in marital status and medical insurance.

**Table 1:** Demographic characteristics of the population with DDM and with UDM in the CHARLS 2011 study.

Characteristics	Patients with DDM	Patients with UDM	p-value
	(n = 958)	(n = 1,285)	
Gender, N (%)			0.004
Male	421 (44.0)	643 (50.0)	
Female	537 (56.0)	642 (50.0)	
Age, N (%)			0.045
45–54 years	230 (24.0)	363 (28.3)	
55–64 years	416 (43.4)	504 (39.2)	
65–74 years	224 (23.4)	280 (21.8)	
≥ 75 years	88 (9.2)	138 (10.7)	
Rural-urban status, N (%)			< 0.001
Rural	421 (44.0)	828 (64.4)	
Urban	537 (56.0)	457 (35.6)	
Regional location, N (%)			< 0.001
East	369 (38.5)	351 (27.3)	
Middle	340 (35.5)	444 (34.6)	
West	249 (26.0)	490 (38.1)	
Highest completed education level, N (%)			< 0.001
Primary school and below	609 (63.6)	906 (70.5)	
Middle school	193 (20.2)	253 (19.7)	
High school and above	156 (16.3)	126 (9.8)	
Marital status, N (%)			0.107
Unmarried/Divorced/Widowed	110 (11.5)	161 (12.5)	
Married	848 (88.5)	1,124 (87.5)	
Occupation, N (%)			< 0.001
Unemployed	44 (4.6)	66 (5.1)	
Agricultural work	444 (46.4)	790 (61.5)	
Employed	150 (15.7)	187 (14.5)	
Self-employed	59 (6.2)	93 (7.2)	
Retired/receded	261 (27.2)	149 (11.6)	
Household personal consumption expenditure, N (%)			< 0.001
Lowest 20%	169 (17.6)	304 (23.7)	
Lower 20%	165 (17.2)	263 (20.5)	
Middle 20%	161 (16.8)	279 (21.7)	
Higher 20%	193 (20.2)	239 (18.6)	
Highest 20%	270 (28.2)	200 (15.6)	
Medical insurance, N (%)			0.522
Uninsured	57 (6.0)	85 (6.6)	
Insured	901 (94.0)	1,200 (93.4)	
Comorbid status, N (%)			< 0.001
No comorbidity	130 (13.6)	383 (29.8)	
1 comorbidity	209 (21.8)	412 (32.1)	
2 comorbidities	246 (25.7)	243 (18.9)	
≥ 3 comorbidities	373 (38.9)	247 (19.2)	

### Direct costs in the population with diabetes

TC and OOP payments are shown in Tables 2 and 3. The median TC and OOP payments were 1,560 CNY (with a mean of 11,193) (US $1,733) and 1,200 CNY (with a mean of 7,266) (US $1,125) for patients with DDM, and 60 CNY (with a mean of 3,700) (US $573) and 24 (with a mean of 3,060) (US $474) for patients with UDM. There was no statistical significance in costs between males and females. Age-related differences were not observed in the population with DDM but were observed in individuals with UDM, since people aged between 55 and 74 years had higher TC than both younger and older individuals (p = 0.039). As to regional differences, urban residents had significantly higher TC and OOP payments than rural residents in the population with DDM (p<0.001, p<0.016). The mean TC and OOP payments were 7,119 and 6,070 CNY (US $1,102 and $940) in rural areas, 14,388 and 8,203 CNY (US $2,227 and $1,270) in urban areas for patients with DDM, 3,934 and 3,237 CNY (US $609 and $501) in rural areas, and 3,276 and 2,740 CNY (US $507 and $424) in urban areas for the population with UDM. Patients living in the west also had significantly higher TC and OOP payments than rural residents in both populations with DDM (p = 0.010, p = 0.003) and with UDM (p<0.001, p<0.001). When grouped by different socio-economic status, the retired/receded had significantly higher and the self-employed had lower TC than patients with other kinds of occupations (p = 0.006) in the population with DDM, while no statistical significance was observed in OOP payments (p = 0.113). However, in the population with UDM, the employed had significantly higher TC and OOP payments than patients with other kinds of occupations (p = 0.013, p = 0.033). No statistical significance was shown for patients with different educational levels and different levels of household personal consumption expenditures. Patients with more kinds of comorbidities also had higher TC and OOP payments, as expected.

**Table 2:** TC and OOP payments in the population with DDM in the CHARLS 2011 study.

Characteristics	Patients with DDM					
	TC Median (Q1, Q3)	TC Mean (SD)	p value	OOP payments Median (Q1, Q3)	OOP payments Mean (SD)	p value
Total	1,560 (24, 7,200)	11,193 (51,300)		1,200 (0, 5,644)	7,266 (21,400)	
Gender			0.960			0.774
Male	1,440 (48, 7,200)	14,756 (70,745)		1,200 (0, 6,000)	7,852 (20,872)	
Female	1,680 (10, 6,960)	8,400 (25,449)		1,200 (0, 5,400)	6,805 (21,813)	
Age			0.708			0.427
45–54 years	1,920 (120, 5,640)	8,390 (20,249)		1,440 (60, 4,818)	7,414 (17,951)	
55–64 years	1,764 (3, 7,380)	10,642 (36,133)		1,332 (0, 6,980)	8,293 (25,923)	
65–74 years	1,200 (20, 7,200)	10,839 (59,910)		1,020 (0, 4,800)	5,893 (17,769)	
≥ 75 years	1,194 (0, 7,200)	22,030 (110,907)		1,072 (0, 5,702)	5,512 (12,710)	
Rural-urban status			**<0.001**			**0.016**
Rural	852 (2, 4,800)	7,119 (18,028)		720 (0, 4,440)	6,070 (15,314)	
Urban	2,400 (72, 8,400)	14,388 (66,490)		1,800 (0, 7,200)	8,203 (25,137)	
Regional location			**0.010**			**0.003**
East	1,200 (0, 4,800)	8,467 (22,149)		720 (0, 4,800)	6,378 (17,556)	
Middle	1,920 (72, 8,400)	8,922 (28,608)		1,680 (24, 6,660)	6,261 (12,159)	
West	1,920 (240, 7,800)	18,334 (90,772)		1,680 (120, 7,200)	9,953 (33,136)	
Highest completed education level			0.094			0.251
Primary school and below	1,200 (24, 6,000)	9,046 (44,578)		1,188 (0, 4,800)	6,119 (13,774)	
Middle school	2,060 (120, 7,200)	15,169 (72,568)		1,800 (36, 5,108)	8,664 (24,338)	
High school and above	2,530 (0, 12,000)	14,657 (43,367)		2,220 (0, 8,204)	10,011 (36,530)	
Marital status			0.158			0.225
Unmarried/ Divorced/ Widowed	1,284 (0, 5,000)	14,279 (83,195)		1,080 (0, 4,800)	7,694 (24,070)	
Married	1,665 (66, 7,200)	10,793 (45,622)		1,200 (0, 5,856)	7,210 (21,044)	
Occupation			**0.006**			0.113
Unemployed	1,560 (348, 3,600)	7,306 (19,334)		1,200 (130, 3,600)	7,096 (19,303)	
Agricultural work	1,164 (0, 5,964)	7,822 (18,432)		846 (0, 4,800)	6,832 (16,337)	
Employed	2,100 (120, 6,000)	7,028 (15,832)		1,512 (48, 4,800)	6,034 (13,914)	
Self-employed	720 (96, 3,600)	3,562 (8,042)		720 (96, 3,600)	3,552 (8,041)	
Retired	2,760 (96, 11,040)	21,703 (93,445)		2,400 (0, 8,024)	9,521 (32,165)	
Household personal consumption expenditure			0.121			0.265
Lowest 20%	800 (48, 4,320)	6,105 (14,721)		612 (26, 3,720)	5,222 (13,047)	
Lower 20%	1,320 (120, 5,122)	8,523 (22,027)		1,200 (72, 4,800)	7,478 (18,907)	
Middle 20%	1,200 (60, 5,520)	7,761 (16,303)		1,200 (0, 4,800)	6,226 (13,151)	
Higher 20%	1,440 (0, 5,312)	6,096 (12,147)		1,200 (0, 4,818)	5,670 (11,936)	
Highest 20%	2,400 (36, 12,000)	21,701 (92,255)		2,253 (0, 7,608)	10,175 (32,967)	
Medical insurance			0.169			0.211
Uninsured	624 (0, 4,320)	4,637 (15,396)		600 (0, 3,600)	4,875 (11,009)	
Insured	1,680 (26, 7,200)	11,494 (52,746)		1,200 (0, 6,000)	7,417 (21,887)	
Comorbid status			**<0.001**			**<0.001**
No comorbidity	552 (0, 3,120)	3,481 (6,839)		480 (0, 2,448)	2,970 (5,875)	
1 comorbidity	852 (0, 4,400)	5,172 (12,473)		720 (0, 3,652)	4,327 (11,466)	
2 comorbidities	1,512 (0, 8,640)	11,026 (24,677)		1,200 (0, 7,200)	7,883 (18,057)	
≥ 3 comorbidities	2,400 (360, 9,600)	17,366 (78,655)		1,920 (240, 7,440)	10,002 (29,307)	

**Table 3:** TC and OOP payments in the population with UDM in the CHARLS 2011 study.

Characteristics	UDM patients					
	TC Median (Q1, Q3)	TC Mean (SD)	p value	OOP payments Median (Q1, Q3)	OOP payments Mean (SD)	p value
Total	60 (0, 1,200)	3,670 (23,824)		24 (0, 1,200)	3,060 (18,654)	
Gender			0.524			0.533
Male	96 (0,1,200)	3,579 (21,770)		60 (0, 1,200)	3,047 (19,580)	
Female	16 (0,1,200)	3,821 (25,734)		0 (0, 1,200)	3,074 (17,693)	
Age			**0.039**			0.081
45–54 years	0 (0, 780)	2,730 (9,576)		0 (0, 720)	2,496 (9,131)	
55–64 years	120 (0, 1,200)	4,319 (35,200)		96 (0, 960)	3,500 (26,786)	
65–74 years	120 (0, 1,817)	4,376 (14,994)		110 (0, 1,728)	3,420 (13,215)	
≥ 75 years	12 (0, 1,560)	2,618 (8,024)		0 (0, 1,360)	2,209 (6,960)	
Rural-urban status			0.689			0.723
Rural	55 (0, 1,200)	3,934 (28,301)		24 (0, 1,200)	3,237 (21,753)	
Urban	60 (0, 1,200)	3,276 (12,057)		24 (0, 960)	2,740 (11,023)	
Regional location			**<0.001**			**<0.001**
East	0 (0, 900)	4,690 (33,820)		0 (0, 720)	3,483 (22,515)	
Middle	12 (0, 1,080)	3,603 (25,529)		0 (0, 744)	3,213 (23,244)	
West	240 (0, 1,800)	3,079 (8,956)		180 (0, 1,500)	2,620 (7,814)	
Highest completed education level			0.485			0.277
Primary school and below	66 (0, 1,260)	2,824 (10,030)		55 (0, 1,200)	2,470 (9,314)	
Middle school	48 (0, 1,200)	7,730 (49,979)		4 (0, 720)	5,910 (37,980)	
High school and above	0 (0, 720)	1,902 (4,955)		0 (0, 720)	1,583 (4,123)	
Marital status			0.375			0.403
Unmarried/ Divorced/ Widowed	0 (0, 1,080)	2,790 (10,942)		0 (0, 1,080)	2,368 (10,282)	
Married	60 (0, 1,200)	3,830 (25,135)		28 (0, 1,200)	3,160 (19,563)	
Occupation			**0.013**			**0.033**
Unemployed	196 (0, 1,500)	3,598 (11,921)		193 (0, 1,500)	3,583 (11,918)	
Agricultural work	96 (0, 1,200)	3,291 (19,889)		66 (0, 1,200)	2,896 (18,059)	
Employed	0 (0, 600)	5,717 (45,244)		0 (0, 576)	4,185 (29,835)	
Self-employed	0 (0, 720)	2,241 (7,953)		0 (0, 720)	2,139 (7,423)	
Retired	120 (0, 2,400)	4,289 (11,549)		24 (0, 1,800)	2,865 (7,913)	
Household personal consumption expenditure			0.465			0.445
Lowest 20%	60 (0, 1,200)	3,699 (28,787)		60 (0, 1,200)	3,302 (2,5982)	
Lower 20%	72 (0, 1,360)	2,380 (9,334)		60 (0, 1,200)	2,008 (8,031)	
Middle 20%	104 (0, 1,200)	2,868 (8,740)		60 (0, 1,200)	2,421 (7,295)	
Higher 20%	0 (0,960)	2,998 (10,027)		0 (0, 720)	2,533 (9,075)	
Highest 20%	78 (0, 1,860)	7,436 (45,177)		0 (0, 1,432)	5,599 (30,832)	
Medical insurance			0.091			0.149
Uninsured	0 (0, 720)	1,968 (6,567)		0 (0, 720)	1,194 (3,195)	
Insured	60 (0, 1,200)	3,823 (24,588)		36 (0, 1,200)	3,193 (19,278)	
Comorbid status			**<0.001**			**<0.001**
No comorbidity	0 (0, 180)	2,437 (31,407)		0 (0, 120)	1,842 (20,664)	
1 comorbidity	0 (0, 720)	2,390 (9,407)		0 (0, 600)	1,958 (8,516)	
2 comorbidities	360 (0, 2,400)	3,276 (8,902)		360 (0, 2,400)	2,563 (6,144)	
≥ 3 comorbidities	1,200 (0, 5,400)	8,260 (34,331)		1,200 (0, 4,800)	7,278 (31,884)	

### Associations between social determinants and medical costs

Results of the two-part model are shown in Tables 4 and 5. In the population with DDM, when adjusted for other factors, being in the 65- to 74- year age group was shown to be negatively associated with TC (p<0.022) and OOP payments (p = 0.010). As to regional factors, living in urban areas was positively associated with TC in patients with DDM (p = 0.048) but negatively associated with both TC and OOP payments in patients with UDM (p = 0.023, p = 0.022). Also, living in the middle or the west was more likely to incur TC (p = 0.023, p = 0.018) and OOP payments (p = 0.011, p = 0.008) in patients with DDM, but was not statistically quantitatively associated with TC and OOP payments. As to socio-economic factors, higher education level was positively associated with TC in patients with DDM, and those who engaged in agricultural work were more likely to incur TC. In addition, higher levels of household personal consumption expenditures were positively associated with TC and OOP payments in the population with UDM and with TC in the population with DDM. It was also noted that married patients with DDM were statistically significantly more likely to incur TC and OOP payments.

**Table 4:** Association between social determinants and TC and OOP in the population with DDM in the CHARLS 2011 study.

Variables	patients with DDM			
	TC		OOP payments	
	Logistic model, OR (95%CI)	Generalized linear model, β (95%CI)	Logistic model, OR (95%CI)	Generalized linear model, β (95%CI)
Gender (ref: male)				
Female	0.921 (0.658, 1.289)	0.051 (-0.239, 0.340)	1.003 (0.726, 1.387)	0.024 (-0.263, 0.311)
Age (ref: 45–54 years)				
55–64 years	0.754 (0.494, 1.149)	0.033 (-0.319, 0.384)	0.735 (0.489, 1.106)	0.007 (-0.341, 0.355)
65–74 years	0.759 (0.458, 1.259)	-0.494 (-0.917, -0.070)*	0.764 (0.469, 1.246)	-0.553 (-0.971, -0.134)*
≥ 75 years	0.731 (0.382, 1.397)	-0.237 (-0.812, 0.338)	0.758 (0.405, 1.422)	-0.524 (-1.092, 0.044)
Rural-urban status (ref: rural)				
Urban	1.045 (0.710,1.537)	0.338 (0.003, 0.672)*	1.022 (0.703, 1.486)	0.285 (-0.047, 0.617)
Regional location (ref: east)				
Middle	1.516 (1.058, 2.172)*	0.105 (-0.213, 0.423)	1.566 (1.107, 2.216)*	0.074 (-0.242, 0.391)
West	1.619 (1.086, 2.414)*	0.193 (-0.148, 0.534)	1.169 (1.150, 2.481)*	0.209 (-0.130, 0.548)
Highest completed education level (ref: primary school and below)				
Middle school	0.974 (0.631, 1.502)	0.080 (-0.286, 0.447)	1.040 (0.684, 1.581)	0.090 (-0.273, 0.454)
High school and above	0.632 (0.388, 1.030)	0.505 (0.067, 0.942)*	0.686 (0.428, 1.097)	0.406 (-0.032, 0.845)
Marital status (ref: unmarried/ divorced/ widowed				
Married	1.884 (1.183, 2.998)*	-0.197 (-0.655, 0.261)	1.708 (1.084, 2.692)*	-0.259 (-0.713, 0.194)
Occupation (ref: unemployed)				
Agricultural work	0.393 (0.155, 0.997)*	0.381 (-0.255, 1.018)	0.578 (0.258, 1.293)	0.167 (-0.478, 0.811)
Employed	0.632 (0.231, 1.728)	0.108 (-0.597, 0.813)	0.986 (0.407, 2.393)	-0.056 (-0.768, 0.656)
Self-employed	0.484 (0.160, 1.467)	-0.359 (-1.162, 0.444)	0.851 (0.311, 2.329)	-0.440 (-1.242, 0.362)
Retired	0.550 (0.209, 1.447)	0.628 (-0.052, 1.308)	0.785 (0.337, 1.829)	0.396 (-0.292, 1.084)
Household personal consumption expenditure (ref: lowest 20%)				
Lower 20%	1.201 (0.699, 2.064)	0.017 (-0.429, 0.463)	1.151 (0.680, 1.948)	0.044 (-0.266, 0.705)
Middle 20%	0.838 (0.789, 1.436)	0.352 (-0.114, 0.818)	0.841 (0.497, 1.425)	0.315 (-0.144, 0.774)
Higher 20%	0.637 (0.380, 1.069)	0.337 (-0.132, 0.805)	0.601 (0.362, 0.997)*	0.387 (-0.075, 0.850)
Highest 20%	0.977 (0.562, 1.697)	0.556 (0.099, 1.033)*	0.804 (0.471, 1.370)	0.460 (-0.004, 0.925)
Medical insurance (ref: uninsured)				
Insured	1.192 (0.623, 2.281)	0.477 (-0.105, 1.060)	1.119 (0.595, 2.104)	0.536 (-0.041, 1.114)
Comorbid status (ref: no comorbidity)				
1 comorbidity	1.079 (0.658, 1.769)	0.223 (-0.264, 0.709)	1.204 (0.743, 1.951)	0.220 (-0.266, 0.705)
2 comorbidities	1.197 (0.739, 1.941)	0.832 (0.363, 1.302)*	1.369 (0.855, 2.193)	0.766 (0.298, 1.234)*
≥3 comorbidities	2.374 (1.464, 3.848)*	0.791 (0.351, 1.232)*	2.239 (1.411, 3.554)*	0.794 (0.352, 1.236)*

*p<0.05.

**Table 5:** Association between social determinants and TC and OOP in the population with UDM in CHARLS 2011 study.

Variables	patients with UDM			
	TC		OOP payments	
	Logistic model, OR (95%CI)	Generalized linear model, β (95%CI)	Logistic model, OR (95%CI)	Generalized linear model, β (95%CI)
Gender (ref: male)				
Female	0.804 (0.625, 1.033)	0.208 (-0.084, 0.499)	0.785 (0.611, 1.009)	0.216 (-0.075, 0.506)
Age (ref: 45–54 years)				
55–64 years	1.288 (0.944, 1.758)	-0.103 (-0.477, 0.271)	1.216 (0.891, 1.659)	-0.154 (-0.528, 0.220)
65–74 years	1.094 (0.752, 1.591)	0.355 (-0.089, 0.799)	1.056 (0.726, 1.535)	0.310 (-0.134, 0.754)
≥ 75 years	0.995 (0.618, 1.604)	0.395 (-0.180, 0.969)	0.926 (0.575, 1.493)	0.351 (-0.227, 0.929)
Rural-urban status (ref: rural)				
Urban	1.214 (0.923, 1.598)	-0.372 (-0.694, -0.050)*	1.181 (0.897, 1.554)	-0.372 (-0.691, -0.053)*
Regional location (ref: east)				
Middle	0.931 (0.687, 1.262)	-0.082 (-0.455, 0.292)	0.880 (0.649, 1.194)	-0.049 (-0.421, 0.323)
West	1.336 (0.990, 1.804)	0.159 (-0.197, 0.514)	1.260 (0.933, 1.700)	0.185 (-0.170, 0.539)
Highest completed education level (ref: primary school and below)				
Middle school	1.158 (0.827, 1.622)	-0.072 (-0.454, 0.310)	1.088 (0.777, 1.522)	-0.138 (-0.523, 0.247)
High school and above	0.901 (0.576, 1.407)	-0.283 (-0.818, 0.252)	0.930 (0.596, 1.452)	-0.262 (-0.799, 0.274)
Marital status (ref: unmarried/ divorced/ widowed				
Married	1.257 (0.863, 1.832)	0.148 (-0.305, 0.600)	1.252 (0.859, 1.825)	0.136 (-0.316, 0.588)
Occupation (ref: unemployed)				
Agricultural work	0.940 (0.538, 1.643)	-0.355 (-0.986, 0.277)	0.905 (0.518, 1.580)	-0.432 (-1.053, 0.189)
Employed	0.622 (0.332, 1.165)	-0.122 (-0.854, 0.611)	0.577 (0.308, 1.081)	-0.167 (-0.892, 0.558)
Self-employed	0.816 (0.409, 1.630)	-0.326 (-1.137, 0.486)	0.784 (0.393, 1.566)	-0.356 (-1.158, 0.445)
Retired	1.016 (0.522, 1.980)	-0.054 (-0.792, 0.683)	0.758 (0.390, 1.473)	-0.176 (-0.914, 0.562)
Household personal consumption expenditure (ref: lowest 20%)				
Lower 20%	0.828 (0.579, 1.184)	0.511 (0.095, 0.926)*	0.846 (0.591, 1.210)	0.468 (0.057, 0.880)*
Middle 20%	0.971 (0.681, 1.383)	0.593 (0.181, 1.005)*	0.966 (0.678, 1.377)	0.563 (0.155, 0.972)*
Higher 20%	0.785 (0.538, 1.145)	0.638 (0.181, 1.095)*	0.808 (0.554, 1.178)	0.524 (0.071, 0.978)*
Highest 20%	0.920 (0.600, 1.410)	0.885 (0.398, 0.372)*	0.844 (0.551, 1.293)	0.775 (0.285, 1.264)*
Medical insurance (ref: uninsured)				
Insured	1.495 (0.928, 2.410)	0.012 (-0.602, 0.627)	1.387 (0.860, 2.237)	0.084 (-0.520, 0.689)
Comorbid status (ref: no comorbidity)				
1 comorbidity	1.883 (1.403, 2.526)*	0.481 (0.077, 0.885)*	1.950 (1.450, 2.622)*	0.322 (-0.085, 0.730)
2 comorbidities	3.332 (2.360, 4.705)*	0.981 (0.555, 1.408)*	3.444 (2.439, 4.861)*	0.921 (0.493, 1.349)*
≥ 3 comorbidities	5.782 (3.993, 8.373)*	1.495 (1.079, 0.911)*	6.151 (4.252, 8.899)*	1.392 (0.975, 1.809)*

*p<0.05.

## Discussion

In our study, we used population-based data from CHARLS to reveal the medical economic burden on middle-aged and elderly individuals with diabetes in China and to study how social determinants were associated with medical costs. Our results showed that the average TC is 11,193 CNY (US $1,733) for patients with DDM and 3,670 CNY (US $568) for patients with UDM, covering 23.6% and 7.7% of GDP per capita in China in 2011, respectively. This result was slightly higher than those reported in Yang’s study because our results included more kinds of comorbidities [29], and lower than those reported in Huang’s study, which was based on the population of a city in east China [30].

### Urban-rurality and medical costs

Regional factors were found to be significantly associated with direct economic costs. First, the TC of patients with DDM and living in urban areas was double that of those living in rural areas. The gap of TC in our study is much narrower than reported in previous studies conducted in a single province of east China [30, 31]. Urban residents incurred an average cost of inpatient visits about 3 times more than the costs incurred by rural residents, while their costs of outpatient visits were similar [20]. The vast difference in the proportion of hospitalization costs was the cause of the large gap of TC between rural and urban areas. The gap in OOP payments, however, was less significant, because urban residents were insured by the urban residents’ basic medical insurance (URBMI), which has higher reimbursement levels than the New Rural Cooperative Medical Insurance (NRCMI), which insures rural residents. The benefits of URBMI would then encourage insured urban residents with DDM to spend more, further widening the gap of TC [30].

To address the urban-rural disparity of economic burden, the urban and rural residents’ basic medical insurance system (URRBMI), which integrated URBMI and NRCMI, has been in effect since 2016. The URRBMI allows rural residents to have the same opportunity of access to medical services and benefit from the same reimbursement levels for medical costs that are equal to those of urban residents [32, 33]. For patients with diabetes, primarily the elderly living in rural areas, URRBMI provided higher levels and higher ceilings for maximum levels of reimbursement than NRCMI, which meant that the elderly then experienced less economic anxiety about incurring healthcare costs at early stages of diabetes [34]. Despite the intent of the URRBMI to accelerate universal health coverage, it did not solve the problem of increasing trends in the occurrence of catastrophic health expenditures [34]. Since the guidelines for diabetes treatment have been updated more frequently in recent years, more new drugs and better therapies are replacing the outdated ones, but these emerging therapeutic approaches are not always included in the list of reimbursements in the medical insurance. Although there will always likely be delays in the insured list, a system that can translate drug price negotiations into timely updates of the insured list must be established to reduce the ascending healthcare costs and stop diabetes-related financial catastrophe.

### Geospatial living status and medical costs

Furthermore, our study found that patients with DDM and living in middle and west China had higher costs of TC and OOP payments, which differed from the comparisons in previous studies [30, 35, 36]. It has been reported that, in the west, people living with diabetes as well as its comorbid diseases such as hypertension, cardiovascular diseases, and arthritis were more seriously affected compared with those living in the east, leading to higher TC and OOP payments [37]. This might explain why our study found that geospatial living status was associated with the incurrence of TC and OOP payments instead of being quantitatively associated with costs, and that patients with DDM and located in middle and west China had higher demand for diabetes-related healthcare services, since they suffered more severe conditions or more complications and comorbidities. This geospatial effect might also account for higher TC and OOP reported by patients with UDM and living in the west; however, their association was not statistically significant when other factors were adjusted in our multivariate model.

A previous study reported that, among the myriad factors contributing to geospatial health disparities, the inequity in health financial investment remained the most outstanding problem, while gaps led by other factors in health resource allocation were decreasing over time [38]. In middle and west China, where economic status was relatively inferior to that in the east, the local government would often focus excessively on economic development and ignore the allocation standards for the same health resources. The shortage of health investment in the west also led to the shortage of better health practitioners and further aggravated the inequity of access to quality in health services [38]. The poorer holistic social environment of public health led to higher health expenditures due to poorer health outcomes, further challenging patients in financial crisis due to diabetes. To break the vicious circle between poverty and serious economic burden, the country launched the ‘poverty alleviation in health’ policy, in which high-quality health resources for those living in the east were funded to serve those in the west. However, this was merely a palliative measure in the long run, and only when the concept that health-related financial investment decreases the economic burden of people takes root in local government leadership under the centralized administrative system in China can this difficult issue be resolved smoothly. Thus, education and administrative training toward developments in areas of public health are needed.

### Socio-economic factors and medical costs

The association between socio-economic factors and medical costs is complex. Patients with DDM who engaged in agricultural work were found to be more likely to incur TC, while the retired/receded had higher TC than did other groups. In China, agricultural workers used to be of relatively poorer socio-economic status, since they were less concerned about their health condition and knew less about diabetes; however, with the changes in lifestyle attributed to economic growth, their risk of diabetes has increased tremendously, such that it could often not be diagnosed in time [39]. Once they were diagnosed, their conditions would often be more serious, leading to increased health expenses. These findings also explain why patients with UDM were less likely to be agricultural workers. In addition, people diagnosed with diabetes were more likely to be retired or receded, probably explaining why patients with DDM were more likely to be retired/receded in our results. These individuals would then incur much higher TC [40, 41]. As to education, patients with DDM who had reached the educational level of high school and above were shown to be positively associated with TC, which was similar to a population-based study which found that the better educated might be in better financial circumstances and spent more on diabetes control [42]. An opposite association was reported in Cai’s study, mainly due to limitations in the study sample [36]. Further, patients with higher household personal consumption expenditure were associated with higher TC and OOP payments, especially in patients with UDM. Wealthier patients usually had more access to health services and were more likely to incur medical costs [28]. For patients with UDM, the wealthier might be more willing to spend on healthcare when they believed it was necessary, even if they did not know they had diabetes, while patients with DDM and in the lower 60% level of household personal consumption expenditure tended to spend to meet the basic demands of diabetes treatment in spite of their financial situation.

In China, the universal coverage of social medical insurance was considered as a solution to improve health equity among people of different socio-economic status, and the New Health Care Reform launched in 2009 was supposed to expand medical insurance coverage to 90% within 3 years [43]. In our study, we found that the medical insurance coverage rate for patients with diabetes had reached 93.7% in 2011. The reform also emphasized the promotion of equalization of basic public health services by establishing a system of personal health records for standardized health-related management of the residents. However, in effect, the quality of public health services was low, for the following reasons: (1) Clinical physicians in China pay little attention to cooperation with public health practitioners, leading to a dearth of clinical participants in disease management; (2) motivational mechanisms, and thus enthusiasm for work, in public health practitioners were weak; (3) the number of public health practitioners remained inadequate, so that the heavy workload was concomitant with a low quality of work [44]. For patients with diabetes, especially those of low socio-economic status, public health management is the most important and effective measure covering primary, secondary, and tertiary prevention to reduce individual healthcare costs. Hence, solutions to address these public health issues should be most immediate for policy-makers.

Also, we found that being in the 65- to 74-year-old age group was negatively associated with TC and OOP payments in patients with DDM. These patients might have suffered from famine exposure in early life, and females often had higher risk for hyperglycemia, while males had lower risk of developing diabetes [45, 46]. Given that our results showed that individuals with DDM were more likely to be male and that patients with UDM and aged 65–74 years had the highest TC, we further analyzed the gender difference for TC in both DDM and UDM patients but found no significance. Thus, this phenomenon remains unexplained, and further study is needed to prove its rationale. Another factor found to be associated with the incurrence of TC and OOP payments in our study and the previous study was marital status, but, until now, there has been limited research on this social mechanism [21].

### Strengths and limitations

Our study was the first to use nationally representative population-based data to reveal the disparities in the direct economic burden of the population with diabetes in China, and was the first to determine how social determinants were directly associated with these disparities in middle-aged and elderly individuals. Compared with previous studies that used hospital-based data or were conducted in specific domestic areas in China, our study provided grander views and more cogent evidence about the direct economic burden on the middle-aged and elderly with diabetes and its association with social determinants. Another advantage of our study was that a significant population with UDM was detected and included for analyses to provide more comprehensive evidence for patients with diabetes overall.

There were several limitations in our study. First, since we used cross-sectional survey data, where cost data were collected retrospectively, information bias and recall bias would inevitably influence their accuracy compared with that of longitudinal data. During the survey, recall bias was generated when patients were asked to recall the number of outpatient and inpatient visits for which they had paid within the recent full year as well as the TC and OOP they spent. Although the investigators asked the interviewees to provide evidence such as hospital receipts to improve data accuracy, the patients’ instinct to protect personal financial privacy, especially for interviewees from rural areas, in fact led to considerable information bias. Moreover, although the study had a nationally-representative sample design, the sample size was relatively small compared with the entire population of China. Also, our study focused only on comparing costs among groups with different social determinants, so we might have inadvertently overlooked the effects of potential physical determinants and confounders on our results [13].

### Recommendations for future research and practice

Based on our findings, policy-makers and healthcare practitioners can take into account the differences in rural-urban and regional status as well as socio-economic status in an attempt to alleviate the economic burden on patients with diabetes. We have proposed potential policy solutions, but future research is needed to evaluate the impact of these policies which focus on the disparities we found in important social determinants. Also, more real-world research is needed to fully characterize the mechanisms of social determinants such as age, gender, and marital status, which were associated with direct costs in patients with diabetes.

## Conclusion

Using nationally representative data, our study revealed the direct economic burden on the Chinese middle-aged and elderly population with diabetes and explains how social determinants were directly associated with these costs. Although health policies in recent years including the implementation of URRBMI, health-related poverty alleviation, and the deepening of the New Health Care Reform, have made progress in containing the growth of the direct economic burden of diabetes, the gaps in TC and OOP payments among populations with different social characteristics remain critical. In the current Chinese setting, health education among local leaders, increasing the proportion of health investment, and improving the quality of public health services are the possible breakthrough points to achieve health equity. Moreover, since early diagnosis was found to be poor in China, health education and community-based screening strategies for diabetes mellitus are crucial actions necessary to improve the prognosis for individuals with diabetes, thereby avoiding considerable economic burden in their later lives.
